# Novel topical allogeneic bone-marrow-derived mesenchymal stem cell treatment of hard-to-heal diabetic foot ulcers: a proof of concept study

**DOI:** 10.1186/s13287-022-02951-8

**Published:** 2022-06-28

**Authors:** Jonas Askø Andersen, Anne Rasmussen, Marie Frimodt-Møller, Susanne Engberg, Esther Steeneveld, Klaus Kirketerp-Møller, Timothy O’Brien, Peter Rossing

**Affiliations:** 1 Diabetes Complications Research, Steno Diabetes Center Copenhagen, Borgmester Ib Juuls Vej 83, 2730 Herlev, Denmark; 2 Orthopedic Department, Nordsjællands Hospital Hilleroed, Dyrehave Vej 2, 3400 Hilleroed, Denmark; 3 Novo Nordisk A/S, Vandtårnsvej 108, 2860 Søborg, Denmark; 4 Leiden University Medical Center, 2300 RC Leiden, The Netherlands; 5 Copenhagen Wound Healing Center Bispebjerg Hospital, Bispebjerg Bakke 23, 2400 Copenhagen, Denmark; 6 Regenerative Medicine Institute CURAM, National University of Ireland Galway, Galway, Ireland; 7 Department of Clinical Medicine, University of Copenhagen, Blegdamsvej 3, 2200 Copenhagen N, Denmark

**Keywords:** Diabetic foot, Mesenchymal stem cells, Clinical trial phase I, Safety

## Abstract

**Aim:**

The aim of this study was to investigate safety of treating diabetic foot ulcers with a topically administered mesenchymal stem cell product.

**Method:**

Individuals with diabetes, peripheral neuropathy, toe blood pressure > 39 mmHg and non-infected foot ulcers with duration of four to fifty-two weeks were screened. Participants were treated with a one-time application of a topically applied allogeneic cellular product containing CD362 enriched mesenchymal stem cells suspended in a collagen solution. Participants were subsequently followed for seven months to gather information on adverse event and serious adverse events.

**Results/discussion:**

A total of sixteen individuals were screened, of whom two were included. The included participants incurred a total of seven adverse events and one serious adverse event. Increased exudation from the treated diabetic foot ulcer was observed for both participants and a connection to investigational medicinal product was suspected. The increased exudation was resolved within one week after application of investigational medicinal product, without any further complications. The serious adverse event consisted of a hospital admission due to neurological symptoms, which were assumed to be caused by hypoglycemia, with no suspected correlation to the investigational medicinal product. None of the other observed adverse events were suspected to be associated with the investigational medicinal product.

**Conclusion:**

This study presents data from two individuals with a diabetic foot ulcer treated with a novel topical mesenchymal stem cell product. An adverse event observed for both participants was suspected to be associated to the investigational medicinal product, i.e., increased exudation, which was resolved within one week, did not lead to further complications and can easily be remedied by choosing bandages with higher absorption capacity or increasing frequency of bandage changes. This study lays the groundwork for further large scale randomized clinical studies.

*Trial registration*: EudraCT number 2015-005580-16. Registered 12/06-2018.

## Background

The lifetime risk of incurring a diabetic foot ulcer (DFU) for an individual with diabetes is 19–34% [1]. The presence of DFUs increase the five-year mortality of the individual that incurs them to that of most common cancers [2] or a 2.5 times higher risk of death than an individual with diabetes without DFUs [3]. Individuals with diabetes harbor a large amount of fear for the consequences of DFUs [4] with good reason as DFUs often lead to infections [5], with a high risk of progressing to an amputation [6].

For society, the burden of DFUs is no less alarming. With a rising population of individuals with diabetes [7] and the cost of treating DFUs on the rise [8, 9].

Several strategies for coping with the challenge of hard-to-heal DFUs have been employed as evidenced by national and international guidelines [10, 11] and several emerging treatments have shown promise including stem cell therapy [12].

The pluripotent nature of stem cells makes them prime candidates for repair of all layers of the healthy epidermis, dermis and subdermal structures that are potentially disturbed by DFUs. The type of stem cell most commonly used in the treatment of DFUs is mesenchymal stem cells (MSCs) [13]. The potential of MSCs to further healing of DFUs has been attributed to the secretion of various cytokines, chemokines and growth factors [13]. In addition, MSCs have been shown to potentially increase neo-angiogenesis which addresses one of the largest challenges when treating DFUs, i.e., tissue hypoxia due to peripheral arterial disease [13]. There are several potential supply sources of MSCs [14–16]. The most commonly used source is bone marrow (BM-MSCs) and there are several randomized clinical studies on systemic and/or injection therapy using BM-MSCs to treat DFUs, but none on topical treatment with BM-MSCs alone [17–23].

The aim of this proof of concept study was to investigate the feasibility and safety of a topically applied allogeneic BM-MSCs seeded in a collagen scaffold for treatment of DFUs.

## Methods

The study was designed as a prospective open label single center proof of concept study.

Between the 1st of August 2018 and the 30th of August 2019 individuals between the ages of 18 and 80 years with type I or II diabetes, no history of cancer within the past five years, no signs of liver disease (i.e. elevated liver function tests or diagnosis of viral hepatitis), HbA1c levels below 97 mmol/mol, peripheral neuropathy and presence of an ulcer below ankle level with a size of 0.25 cm^2^ to 7.5 cm^2^ and duration of 4 to 52 weeks before inclusion were screened at Steno Diabetes Center Copenhagen (SDCC).

Screened individuals were excluded if toe blood pressure was found to be below 40 mmHg, if there were signs of active Charcot arthropathy, signs of infection in the treated DFUs or the treated extremity and if screened individuals had received treatment with systemic or local therapies that could affect healing of the DFUs or the effects of the investigational medicinal product (IMP) (i.e. corticosteroids, biological medicine or chemotherapy) within 30 days prior to inclusion. In addition, individuals were excluded if they had undergone surgery that could affect the DFUs healing potential (i.e. Achilles tendon lengthening or vascular surgery) within three months prior to inclusion.

Individuals were identified in the foot clinic at SDCC by the treating podiatrist. Relevant individuals were subsequently screened and informed by educated scientific staff.

Individuals that met all in- and none of the exclusion criteria were enrolled in the study, with a run-in period where DFUs were observed for potential healing. Only DFUs that did not heal during the run-in period were included. After the run-in period, treatment with IMP was applied once, and participants were observed for six months.

In the observation period after treatment, the participants were initially seen on a weekly basis until week 12, after which participants were seen at week 14, 16, 20 and 24.

A safety board of experts monitored the study and were consulted on all serious adverse events (SAEs) that were observed in the study.

The aim of the study was to evaluate the safety of the IMP, which was done by monitoring incidence and causality of adverse events (AEs) and SAEs observed in the study. Hence, the study was not powered to examine ulcer healing.

The study was initially planned to include nine participants, but due to challenges in recruitment, the project was ended before this goal was reached.

### Definitions

Definition of DFUs were; lesions of the skin of the feet of an individual with diabetes [24].

Healed DFUs were defined as; intact skin completely covered with intact epithelium [25].

Peripheral neuropathy was defined as; vibration sensation (measured by biothesiometri) > 24 V and/or absent sensation to monofilament.

Peripheral arterial disease (PAD) was graded according to the wound, ischemia and foot infection classification [26]. PAD was graded as:

Grade 0 (normal) toe pressure > 60 mmHg.

Grade 1—40–59 mmHg.

Grade 2—30–39 mmHg.

Grade 3—< 30 mmHg.

### Measurements and interventions

#### Investigational medicinal product formulation and intervention

The IMP was manufactured at Interdivisional good manufacturing practice (GMP) Facility, Department of Clinical Pharmacy and Toxicology, Leiden University Medical Center, Leiden, Netherlands (LUMC), according to EU Guidelines on GMP, IMP.

The IMP was an allogeneic cellular therapy product containing CD362 enriched BM-MSCs from adult bone marrow (REDDSTAR ORBCEL-M, Orbsen Therapeutics Ltd. Galway, Ireland). Bone marrow was acquired from healthy non-related and leukocyte antigen unmatched donors. The MSCs were suspended in a HypoThermosol formulation (Biolife Solutions Inc. Bothell, Washington, USA).

When a participant was booked for a treatment visit after completed run-in period, message to prepare an IMP dose was send to LUMC. After receiving the request, a single dose of the IMP (30 × 10^6^ BM-MSCs) was thawed, expanded, centrifuged and resuspended in 500 µl HypoThermosol (Biolife Solutions, Bothell, WA, USA) in a 1 ml syringe. The BM-MSCs product was then send to the treatment facility.

**Fig. 1:**
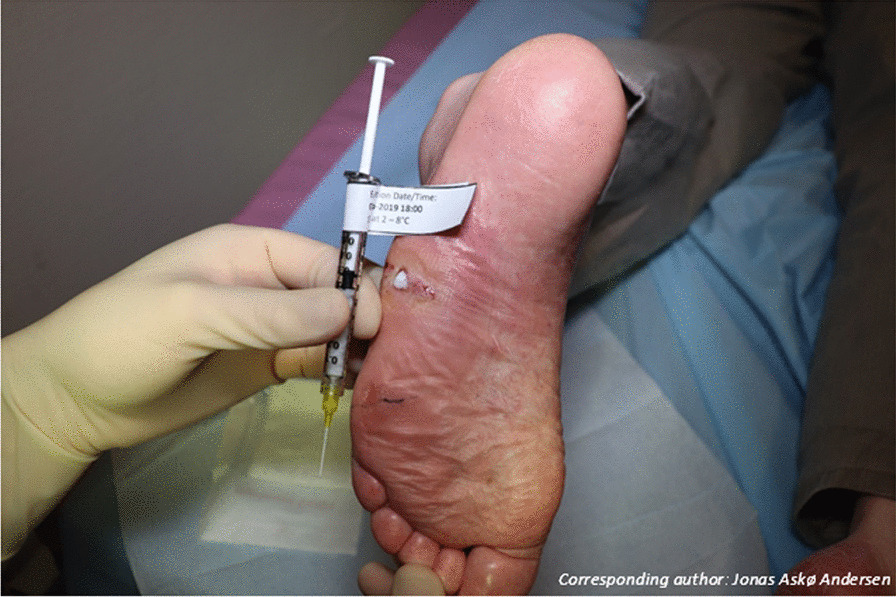
Clinical photo of investigational medicinal product application

Before application of IMP, DFUs was debrided and cleaned according to standard procedure. The BM-MSCs formulation was mixed with a 6.5% collagen solution (Collagen Solutions plc. London, UK) on site before application. When ready for application, a 1 ml syringe containing 0.35 ml 6.5% collagen was fitted to a 1 ml syringe containing 0.5 mL of the BM-MSCs solution via a Luer lock connector and mixed, in a biosafety cabinet, by passing the syringe back and forth 12 times, with a final homogenous 2.6% Collagen BM-MSCs solution. The final product was then transported to the patient in a tamper-proof container prior to administration. The dose administered was calculated according to the area of the debrided ulcer with 10.6 × 10^6^ BM-MSCs/3 cm^2^ as the target dose. Using a 1 ml syringe and a 24 gauge catheter, the formulation was applied directly to debrided and cleaned ulcer as a film that covered the entire ulcer (Fig. 1) and the formulation and ulcer were subsequently covered with a foam bandage, three layers of gauze and fixed with an elastic bandage.

#### Standard treatment

At all visits, standardized care was performed including; ulcer assessment, ulcer debridement and cleaning, callus trimming, application of bandages, in addition to control, adjustment and application of offloading devices.

By use of a novel 3D laser technology, the ulcer was measured after debridement, including area, perimeter, depth and volume at all visits (Silhouette™ camera (Aranz-medical, Christchurch, New Zealand)) and analyzed using the Silhouette™ software. The area of the ulcer was noted in mm^2^ and the depth of the ulcer was extracted from the used software and noted.

In the following text, all measurements of DFUs refer to those obtained from the Silhouette™ camera.

## Results

### Study-population

**Fig. 2:**
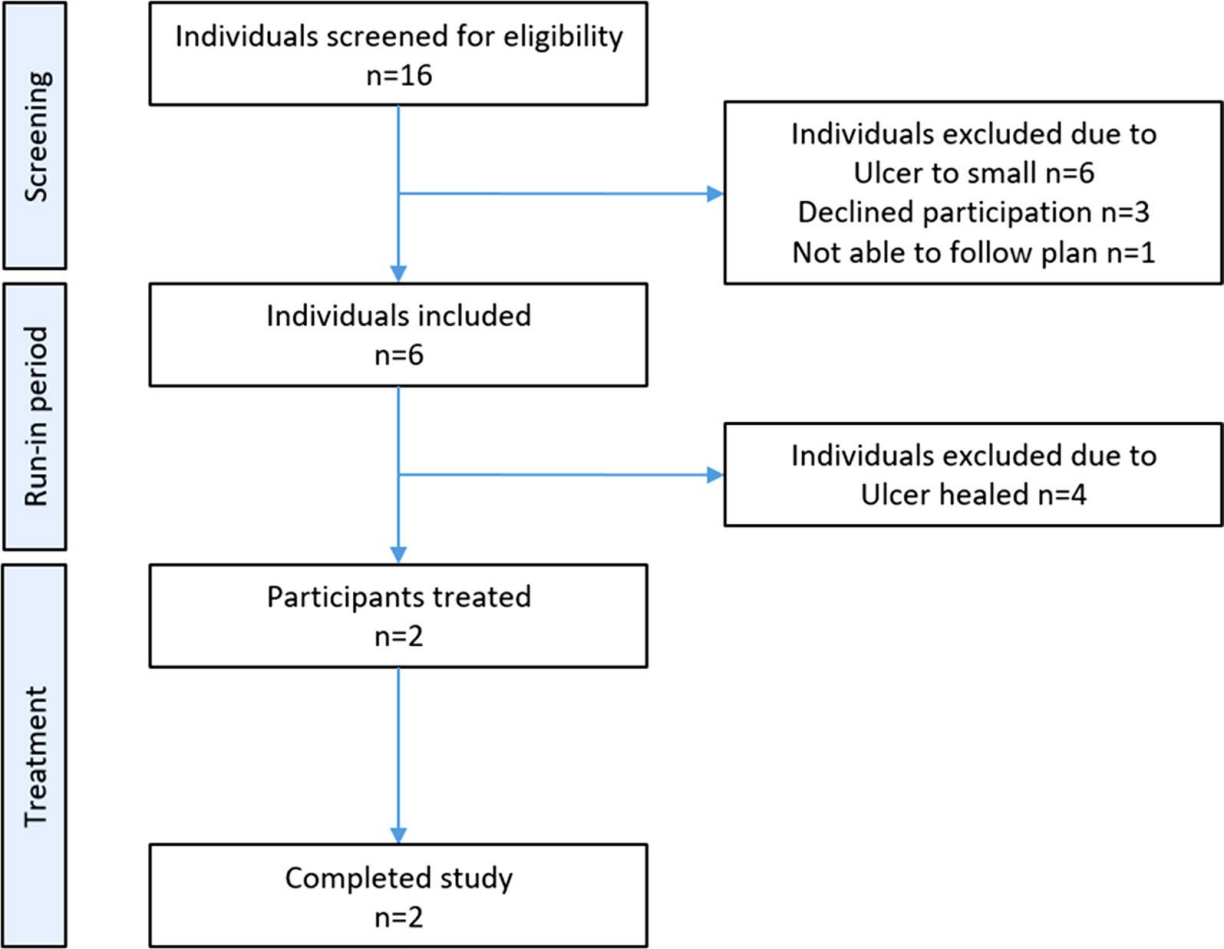
Consort diagram

Of the 16 identified candidates, six were included but only two fulfilled the inclusion and exclusion criteria for intervention and completed the study (Fig. 2). Both participants had type II diabetes with a diabetes duration of 12 and six years respectively (Table 1).

**Table 1:** Participant characteristics

	Age (years)	Diabetes type	Diabetes duration (years)	BMI (kg/m^2^)	Smoker^a^	Alcohol abuse	Sys. blood pressure (mmHg)	HbA1c^b^ (mmol/ml)	eGFR (ml/min)	UACR (mg/g)	LDL (mmol/l)
Participant 01	70	T2DM	12	23.9	No	No	150	57	83	27	1.8
Participant 02	68	T2DM	6	29.0	Ex	No	108	93	99	20	0.3

BMI, Body Mass Index; Sys, Systolic; HbA1C, Glycated hemoglobin; eGFR, estimated Glomerular Filtration Rate; UACR, Urine-Albumin-Creatinin-Ratio; LDL. Low density Lipoprotein; T2DM, Type II Diabetes; Mellitus; Ex, Former

^a^Divided in Yes(active) No (never smoked) Ex. (prior smoker > 5 years tobacco free)

^b^HbA1c is presented according to National Glycohemoglobin Standardization Program

### Clinical outcomes

#### Participant 01

The index ulcer was placed on the lateral aspect of the foot corresponding to the proximal prominence of the fifth metatarsal. Ulcer duration prior to inclusion was 47 weeks.

Participant 01 had a total of 21 visits during the follow-up (one extra visit, compared to protocol). Participant 01 had peripheral neuropathy and no PAD (grade 0) (Table 2).

**Table 2:** Ulcer details

	Vib sens. (V) (right/left)	Monofilament Sensation^a^ (right/left)	Palpable Foot pulses^b^ (right/left)	TBP (mmHg) (right/left)	Follow-Up (weeks)	Ulcer Duration (weeks)	Prior amputations	Offloading
Participant 01	37/42	Neg/Neg	Yes/Yes	84/79	31	47	Yes (partial toe)	Yes Air-cast Rocker bottom sandals
Participant 02	50/50	Pos/Pos	Yes/Yes	46/76	27	7	Yes (trans-metatarsal toe)	Yes Rocker bottom sandals Handmade shoes

*Vib sens*, Vibration sensation measured by biothesiometri; TBP, Toe Blood Pressure

^a^Monofilament performed with 10 g Siemens Weinstein test. Pos(itive) = no sensation, Neg(ative) = intact sensation

^b^Palpable foot pulses defined as palpable pulse in arteria tibialis posterior and/or arteria dorsalis pedis

**Fig. 3:**
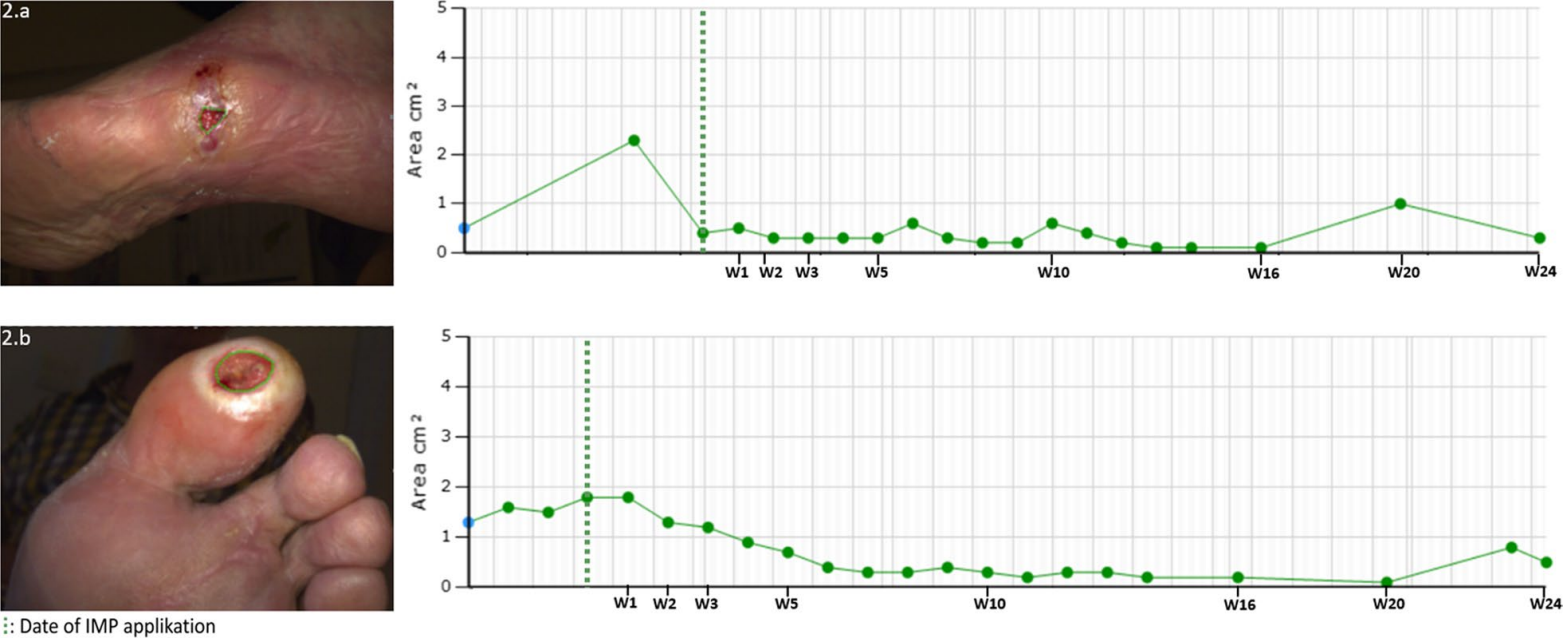
Clinical photo of index ulcer and graph showing progression of ulcer area. 2.a: Participant 01, 2.b: Participant 02. W: weeks from date of IMP application, IMP: Investigational medicinal product

The ulcer area was 50 mm^2^ at inclusion and 30 mm^2^ at study end but fluctuated during the observation period (Fig. 3), with the largest area of 230 mm^2^, and smallest area of 10 mm^2^. The ulcer did not completely heal during follow-up.

Participant 01 incurred four AEs and no SAEs during the observation period. Of the incurred AEs, one was suspected to be associated with the IMP.

Participant 01 was seen one week after application of BM-MSCs formulation due to increased exudation from the treated ulcer. A connection between the AE and the IMP was suspected. The increased exudation was resolved at the following visit, one week later, did not result in worsening of the index ulcer and did not reoccur.

In addition, participant 01 experienced increased symptoms of allergic rhinitis two months after treatment. The diagnosis of allergic rhinitis had been made prior to participating in this study. The increased allergic symptoms were not suspected to be attributed to the IMP and receded after treatment with oral antihistamine treatment.

At the visit three months after IMP application, dry skin in both palms and in a small area on the medial side of the right elbow was noted. The dry skin disappeared after treatment with moisturizing crème. The dry skin was not suspected to be attributed to the IMP.

At the visit four months after treatment, the patient had incurred a superficial infection of the index ulcer. The infection was treated with oral antibiotics for ten days and was resolved at the visit one week later. The infection was not suspected to be attributed to the IMP.

#### Participant 02

The index ulcer was placed on pulpa of the left first toe. Ulcer duration prior to inclusion was seven weeks.

Participant 02 had a total of 22 visits during the follow-up (three extra visits, compared to protocol), the discrepancy in number of visits is explained by an amendment to the protocol being approved and implemented between the two participants treatment and observation periods. Participant 02 had peripheral neuropathy and PAD (grade 1) (Table 2).

The ulcer area was 130 mm^2^ at inclusion and 50 mm^2^ at end of study but fluctuated during the observation period (Fig. 3), with largest area of 540 mm^2^, and smallest area of 10 mm^2^. The ulcer did not completely heal during follow-up.

Participant 02 incurred three AEs and one SAE during the observation period. Of the incurred AEs, one was suspected to be associated with the IMP.

Similar to participant 01, participant 02 experienced increased exudate from index ulcer after application of BM-MSCs formulation. One week after application of BM-MSCs formulation participant 02 was seen in the clinic for an extra visit due to increased exudation from the index ulcer, which was suspected to be related to the IMP. The increased exudation was resolved at the following visit one week later, did not result in worsening of the index ulcer and did not reoccur.

Participant 02 was admitted to the hospital two months after BM-MSCs application due to changed cerebral status with slurred speech and confusion. All tests, including lumbar puncture, computed tomography and magnetic resonance scan of the cerebrum were normal. The incident was assumed to be related to a case of hypoglycemia. There was no recurrence of symptoms, and the patient recovered fully. The incident was noted as an SAE and no association to the IMP was suspected.

Following the SAE, participant 02 incurred bullae on the dorsal aspect of both feet, in accordance with the diagnosis of bullosis diabeticorum. The bullaes healed without complications within four weeks. The presumed bullosis diabeticorum was not suspected to be attributed to the IMP. Five months after treatment, participant 02 incurred an infection of the index ulcer with increased size and fetid odor. The infection was treated with oral antibiotics for 10 days and was resolved at the visit one week later. No connection between the infection and the IMP was suspected.

## Discussion

In this study, we present data on safety and tolerability of a novel topical treatment with BM-MSCs applied to DFUs of two individuals.

None of the two index DFUs healed during the follow-up. However, this study was not designed to evaluate healing of DFUs, but rather to gauge safety profile of the novel BM-MSCs treatment.

The most common tissue to extract MSCs for the treatment of DFUs is bone marrow. In Table 3, the published randomized clinical trials on BM-MSCs treatment of DFUs are presented. As seen most studies on treatment with BM-MSCs of DFUs have focused on autologous treatment where cells are extracted from the treated individual. Although autologous stem cell treatment has shown promise several potential challenges remain before clinical implementation in the treatment of DFUs can be realized. Diabetes is known to have a detrimental effect on MSCs count and potency [27] and it is plausible that other diseases, medical treatments, radiation therapy, etc. could have an effect on the potency and amount of MSCs harvested from bone marrow. Secondly, the patients are subjected to several risks when performing the harvesting, i.e., placing patients in a hypercoagulable state, performing an invasive procedure, and exposing patients to epidural procedure and sedation. Finally, autologous harvesting of BM-MSCs requires a large setup, both for the harvesting procedure and the subsequent preparation and suspension of the cells, which will inevitably reduce implementation of this treatment in daily practice.

**Table 3:** Overview of randomized clinical studies on bone-marrow-derived stem cell treatment of diabetic foot ulcers published within the last ten years

Author	Method	Participants	Formulation^a^	Method of delivery	Outcomes
Debin et al. [17]	Patients randomized to BMMSC or standard care	38	Autologous BMMSC	Intramuscular and intralesional injections	Increased complete ulcer healing rates in treatment group Increased ABI in treatment group Decreased amputation rates
Dash et al. [18]	Patients randomized to BMMSC or standard care	6	Donor BMMSC	Intramuscular injections, intralesional injections and topical application	Increased ulcer healing rates
Prochazka et al. [23]	Patients randomized to BMMSC or standard care	96	Autologous BMMSC	Intramuscular injections	Decreased amputation rates in treatment group
Lu et al. [21]	Patients randomized to BMMSC or BMMNC in one limb and saline in the other limb	41	Autologous BMMSC Autologous BBMNC	Intramuscular and intralesional injections	Increased complete ulcer healing *BMMSC* > *BMMNC* > *NS* Decreased amputation rates *BMMSC* > *BMMNC* > *NS*
Jain et al. [19]	Patients randomized to BMMSC or whole Blood	48	Autologous BMMSC	Intralesional injection and topical application	Increased complete ulcer healing in treatment group^b^
Kirana et al. [20]	Patients randomized to BMMSC or BMMNC in both limbs	22	Autologous BBMSC Autologous BBMNC	Intramuscular injection or Intraarterial injection	Improved TcPO_2_ from baseline in both groups No significant difference in ulcer healing
Lu et al. [22]^c^	Patients randomized to BMMSC or BMMNC in one limb and saline in the other limb	41	Autologous BMMSC Autologous BBMNC	Intramuscular and intralesional injections	Decreased incidence of ulcer recurrence for BMMSC (vs. NS) No significant difference in amputation rates

BMMSC, bone marrow mesenchymal stem cells; ABI, ankle-brachial-index; BMMNC, bone marrow-derived mononuclear cells; NS, normal saline; TcPO_2_, transcutaneous partial pressure of oxygen

^a^Refers to the applied cell types. However, the preparation and suspension of cells differed from study to study

^b^Complete ulcer healing was defined as both healed by secondary intention and treated with skin graft

^c^Follow-up study on patients from 2011 study by Lu et al

If the treatment of DFUs with stem-cell treatment is to be part of the everyday clinic, products like the IMP investigated in this study has the potential to solve some of the above-mentioned challenges. The IMP is donated by healthy donors, preparation and suspension of cells are performed before delivery and application is topical with no need for invasive procedures or additional medical treatment. As of now there are no commercially available products with BM-MSCs for DFUs available, leaving treatment with BM-MSCs to experimental and specialized centers which are not accessible to the general population with DFUs.

Only one study was found, reporting on systemic complications following topical treatment with BM-MSCs. Although the treatment consisted of simultaneous systemic and local injection therapy combined with topical application [18]. Other studies have examined the safety of topically applied adipose tissue derived MSCs and did not report any AEs associated to the investigated treatment [28, 29]. However, no studies have reported on safety or efficacy when treating DFUs with topical BM-MSCs alone.

Phase I and II trials on MSCs treatment of diseases as osteoarthritis, cardiac insufficiency, myocardial infarction and limb ischemia have not found any AEs related to MSCs treatment [30–32]. It has been suggested that MSCs could potentially proliferate to tumors and contribute to formation of tumor stroma [33]. Although this suspicion has not been confirmed, one case study reported findings of angio-myeloproliferative lesions in a kidney that was subjected to injections with hematopoietic stem cells and a connection was suspected [34].

In the randomized clinical studies listed in Table 3, none reported any AEs related to treatment with BM-MSCs [17–23].

In the current study, we found one AE that both study participants experienced, i.e., short-term increased exudate from the treated ulcer. The AE could be associated to the treatment with the IMP and is potentially serious, as increased exudate can lead to maceration, which is detrimental to ulcer healing. In both cases, the AE was resolved within one week and can easily be remedied by either choosing other bandages with higher absorption capacity or changing bandages more frequently after application of IMP. No further AEs or SAEs suspected to be associated to the IMP were observed.

There are limitations to this study; first and foremost, the low number of participants recruited for the study. The rigid in- and exclusion criteria reduced pool of candidates and the number of visits discouraged some of the relevant candidates. In addition, the run in period for newly diagnosed DFUs resulted in likely candidates being excluded due to healing of ulcers before application of IMP. Although this study adds to the growing evidence that treatment with BM-MSCs could be part of the solution to the growing global challenge that is DFUs it only identifies a potential candidate that needs further investigations in future randomized studies. Future studies should focus on clinical outcomes such as ulcer healing and compare effects of topical applied BM-MSCs to treatments known to be effective in the treatment of DFUs like autologous combined leucocyte, platelet and fibrin products [35].

## Conclusion

While a common AE in the form of increased exudation from the treated ulcer was found to be associated with the IMP, the AE was easily remedied and did not have long-term implications for the treated individuals. With this in mind this small study supports that this novel topically applied BM-MSCs formulation is a safe treatment option for DFUs. It is, however, accepted that only two patients were treated and thus the study might be considered a “proof of concept study”.

## Data Availability

The data that support the findings of this study are available on request from the corresponding author (Jonas Askø Andersen) pending acceptance from Danish Data Protection Agency. The data are not publicly available due to privacy or ethical restrictions.

## Abbreviations

AE: Adverse event
BM-MSC: Bone marrow mesenchymal stem cell
DFU: Diabetic foot ulcer
IMP: Investigational medicinal product
MSC: Mesenchymal stem cell
PAD: Peripheral arterial disease
SDCC: Steno diabetes center Copenhagen

## References

[1] Everett E, Mathioudakis N (2018). Update on management of diabetic foot ulcers. Ann N Y Acad Sci.

[2] Armstrong DG, Swerdlow MA, Armstrong AA, Conte MS, Padula WV, Bus SA (2020). Five year mortality and direct costs of care for people with diabetic foot complications are comparable to cancer. J Foot Ankle Res.

[3] Walsh JW, Hoffstad OJ, Sullivan MO, Margolis DJ (2016). Association of diabetic foot ulcer and death in a population-based cohort from the United Kingdom. Diabet Med.

[4] Vileikyte L, Pouwer F, Gonzalez JS (2020). Psychosocial research in the diabetic foot: are we making progress?. Diabetes Metab Res Rev.

[5] Prompers L, Huijberts M, Apelqvist J, Jude E, Piaggesi A, Bakker K (2007). High prevalence of ischaemia, infection and serious comorbidity in patients with diabetic foot disease in Europe Baseline results from the Eurodiale study. Diabetologia.

[6] Lipsky BA, Berendt AR, Cornia PB, Pile JC, Peters EJ, Armstrong DG (2012). Infectious Diseases Society of America clinical practice guideline for the diagnosis and treatment of diabetic foot infections. Clin Infect Dis.

[7] WHO. Diabetes Key Facts. 2020.

[8] Raghav A, Khan ZA, Labala RK, Ahmad J, Noor S, Mishra BK (2018). Financial burden of diabetic foot ulcers to world: a progressive topic to discuss always. Ther Adv Endocrinol Metab.

[9] Tchero H, Kangambega P, Lin L, Mukisi-Mukaza M, Brunet-Houdard S, Briatte C (2018). Cost of diabetic foot in France, Spain, Italy, Germany and United Kingdom: a systematic review. Ann Endocrinol (Paris).

[10] Parker CN, Van Netten JJ, Parker TJ, Jia L, Corcoran H, Garrett M (2019). Differences between national and international guidelines for the management of diabetic foot disease. Diabetes Metab Res Rev.

[11] Schaper NC, van Netten JJ, Apelqvist J, Bus SA, Hinchliffe RJ, Lipsky BA (2020). Practical guidelines on the prevention and management of diabetic foot disease (IWGDF 2019 update). Diabetes Metab Res Rev.

[12] Ramirez-Acuña JM, Cardenas-Cadena SA, Marquez-Salas PA, Garza-Veloz I, Perez-Favila A, Cid-Baez MA (2019). Diabetic foot ulcers: current advances in antimicrobial therapies and emerging treatments. Antibiotics (Basel)..

[13] An T, Chen Y, Tu Y, Lin P (2020). Mesenchymal stromal cell-derived extracellular vesicles in the treatment of diabetic foot ulcers: application and challenges. Stem Cell Rev Rep..

[14] Qin HL, Zhu XH, Zhang B, Zhou L, Wang WY (2016). Clinical evaluation of human umbilical cord mesenchymal stem cell transplantation after angioplasty for diabetic foot. Exp Clin Endocrinol Diabetes.

[15] Vojtassák J, Danisovic L, Kubes M, Bakos D, Jarábek L, Ulicná M (2006). Autologous biograft and mesenchymal stem cells in treatment of the diabetic foot. Neuro Endocrinol Lett.

[16] Horie T, Yamazaki S, Hanada S, Kobayashi S, Tsukamoto T, Haruna T (2018). Outcome from a randomized controlled clinical trial - improvement of peripheral arterial disease by granulocyte colony-stimulating factor-mobilized autologous peripheral-blood-mononuclear cell transplantation (IMPACT). Circ J.

[17] Debin L, Youzhao J, Ziwen L, Xiaoyan L, Zhonghui Z, Bing C (2008). Autologous transplantation of bone marrow mesenchymal stem cells on diabetic patients with lower limb ischemia⋆ ⋆Supported by the Clinical Research Fund of Southwest Hospital at Third Military Medical University (SWH2005A109). J Med Colleges PLA.

[18] Dash NR, Dash SN, Routray P, Mohapatra S, Mohapatra PC (2009). Targeting nonhealing ulcers of lower extremity in human through autologous bone marrow-derived mesenchymal stem cells. Rejuvenation Res.

[19] Jain P, Perakath B, Jesudason MR, Nayak S (2011). The effect of autologous bone marrow-derived cells on healing chronic lower extremity wounds: results of a randomized controlled study. Ostomy Wound Manag.

[20] Kirana S, Stratmann B, Prante C, Prohaska W, Koerperich H, Lammers D (2012). Autologous stem cell therapy in the treatment of limb ischaemia induced chronic tissue ulcers of diabetic foot patients. Int J Clin Pract.

[21] Lu D, Chen B, Liang Z, Deng W, Jiang Y, Li S (2011). Comparison of bone marrow mesenchymal stem cells with bone marrow-derived mononuclear cells for treatment of diabetic critical limb ischemia and foot ulcer: a double-blind, randomized, controlled trial. Diabetes Res Clin Pract.

[22] Lu D, Jiang Y, Deng W, Zhang Y, Liang Z, Wu Q (2019). Long-term outcomes of BMMSC compared with BMMNC for treatment of critical limb ischemia and foot ulcer in patients with diabetes. Cell Transplant.

[23] Procházka V, Gumulec J, Jalůvka F, Salounová D, Jonszta T, Czerný D (2010). Cell therapy, a new standard in management of chronic critical limb ischemia and foot ulcer. Cell Transplant.

[24] Health DNBo. Diabetic foot ulcers—a health technology assessment. Copenhagen: Danish National Board of Health; 2011.

[25] Almdal T, Nielsen AA, Nielsen KE, Jorgensen ME, Rasmussen A, Hangaard S (2015). Increased healing in diabetic toe ulcers in a multidisciplinary foot clinic—an observational cohort study. Diabetes Res Clin Pract.

[26] Conte MS, Bradbury AW, Kolh P, White JV, Dick F, Fitridge R (2019). Global vascular guidelines on the management of chronic limb-threatening ischemia. J Vasc Surg.

[27] Zhou B, Bi YY, Han ZB, Ren H, Fang ZH, Yu XF (2006). G-CSF-mobilized peripheral blood mononuclear cells from diabetic patients augment neovascularization in ischemic limbs but with impaired capability. J Thromb Haemost.

[28] Moon KC, Suh HS, Kim KB, Han SK, Young KW, Lee JW (2019). Potential of allogeneic adipose-derived stem cell-hydrogel complex for treating diabetic foot ulcers. Diabetes.

[29] Carstens MH, Quintana FJ, Calderwood ST, Sevilla JP, Ríos AB, Rivera CM (2021). Treatment of chronic diabetic foot ulcers with adipose-derived stromal vascular fraction cell injections: safety and evidence of efficacy at 1 year. Stem Cells Transl Med.

[30] Philippe B, Luc S, Valérie PB, Jérôme R, Alessandra BR, Louis C (2010). Culture and use of mesenchymal stromal cells in phase I and II clinical trials. Stem Cells Int.

[31] Emadedin M, Labibzadeh N, Liastani MG, Karimi A, Jaroughi N, Bolurieh T (2018). Intra-articular implantation of autologous bone marrow-derived mesenchymal stromal cells to treat knee osteoarthritis: a randomized, triple-blind, placebo-controlled phase 1/2 clinical trial. Cytotherapy.

[32] Bura A, Planat-Benard V, Bourin P, Silvestre JS, Gross F, Grolleau JL (2014). Phase I trial: the use of autologous cultured adipose-derived stroma/stem cells to treat patients with non-revascularizable critical limb ischemia. Cytotherapy.

[33] Hall B, Andreeff M, Marini F. The participation of mesenchymal stem cells in tumor stroma formation and their application as targeted-gene delivery vehicles. Handb Exp Pharmacol. 2007:263–83. 10.1007/978-3-540-68976-8_12 17554513

[34] Thirabanjasak D, Tantiwongse K, Thorner PS (2010). Angiomyeloproliferative lesions following autologous stem cell therapy. J Am Soc Nephrol.

[35] Game F, Jeffcoate W, Tarnow L, Jacobsen JL, Whitham DJ, Harrison EF (2018). LeucoPatch system for the management of hard-to-heal diabetic foot ulcers in the UK, Denmark, and Sweden: an observer-masked, randomised controlled trial. Lancet Diabetes Endocrinol.

[36] Use TICfHoTRfPfH. E6(R2) Guideline “Good Clinical Practice” (GCP). 2021.

